# Gene Expression Signatures Can Aid Diagnosis of Sexually Transmitted Infection-Induced Endometritis in Women

**DOI:** 10.3389/fcimb.2018.00307

**Published:** 2018-09-20

**Authors:** Xiaojing Zheng, Catherine M. O'Connell, Wujuan Zhong, Taylor B. Poston, Harold C. Wiesenfeld, Sharon L. Hillier, Maria Trent, Charlotte Gaydos, George Tseng, Brandie D. Taylor, Toni Darville

**Affiliations:** ^1^Department of Pediatrics, University of North Carolina at Chapel Hill, Chapel Hill, NC, United States; ^2^Department of Biostatistics, University of North Carolina at Chapel Hill, Chapel Hill, NC, United States; ^3^Department of Obstetrics, Gynecology and Reproductive Sciences, Magee-Womens Research Institute, Pittsburgh, PA, United States; ^4^Department of Obstetrics, Gynecology, and Reproductive Sciences, University of Pittsburgh, Pittsburgh, PA, United States; ^5^Section on Adolescent Medicine, Department of Pediatrics, Johns Hopkins School of Medicine, Johns Hopkins University, Baltimore, MD, United States; ^6^Division of Infectious Diseases, Department of Medicine, Johns Hopkins School of Medicine, Johns Hopkins University, Baltimore, MD, United States; ^7^Department of Biostatistics, University of Pittsburgh, Pittsburgh, PA, United States; ^8^Department of Epidemiology and Biostatistics, Texas A&M University, College Station, TX, United States

**Keywords:** biomarker, mRNA, Chlamydia, gonorrhea, pelvic inflammatory disease

## Abstract

Sexually transmitted infection (STI) of the upper reproductive tract can result in inflammation and infertility. A biomarker of STI-induced upper tract inflammation would be significant as many women are asymptomatic and delayed treatment increases risk of sequelae. Blood mRNA from 111 women from three cohorts was profiled using microarray. Unsupervised analysis revealed a transcriptional profile that distinguished 9 cases of STI-induced endometritis from 18 with cervical STI or uninfected controls. Using a hybrid feature selection algorithm we identified 21 genes that yielded maximal classification accuracy within our training dataset. Predictive accuracy was evaluated using an independent testing dataset of 5 cases and 10 controls. Sensitivity was evaluated in a separate test set of 12 women with asymptomatic STI-induced endometritis in whom cervical burden was determined by PCR; and specificity in an additional test set of 15 uninfected women with pelvic pain due to unknown cause. Disease module preservation was assessed in 42 women with a clinical diagnosis of pelvic inflammatory disease (PID). We also tested the ability of the biomarker to discriminate STI-induced endometritis from other diseases. The biomarker was 86.7% (13/15) accurate in correctly distinguishing cases from controls in the testing dataset. Sensitivity was 83.3% (5/6) in women with high cervical *Chlamydia trachomatis* burden and asymptomatic endometritis, but 0% (0/6) in women with low burden. Specificity in patients with non-STI-induced pelvic pain was 86.7% (13/15). Disease modules were preserved in all 8 biomarker predicted cases. The 21-gene biomarker was highly discriminatory for systemic infections, lupus, and appendicitis, but wrongly predicted tuberculosis as STI-induced endometritis in 52.4%. A 21-gene biomarker can identify asymptomatic women with STI-induced endometritis that places them at risk for chronic disease development and discriminate STI-induced endometritis from non-STI pelvic pain and other diseases.

## Introduction

Pelvic inflammatory disease (PID) occurs when sexually transmitted pathogens ascend from the cervix to the uterus and oviducts, resulting in endometritis and salpingitis. Infection with *Chlamydia trachomatis* (CT), *Neisseria gonorrhoeae* (GC), or both, may lead to PID and associated morbidities including infertility, ectopic pregnancy, and chronic pelvic pain. *Mycoplasma genitalium* has emerged as a pathogen causing PID and its associated long-term complications (McGowin and Anderson-Smits, 2011; Bjartling et al., 2012). PID affects approximately 800,000 women annually in the United States (Centers for Disease Control Prevention., 2017), and yearly medical expenditures for PID treatment approach ~2 billion dollars (Rein et al., 2000).

The diagnosis of PID is imprecise. No single historical, physical, or laboratory finding is both sensitive and specific for the diagnosis of PID (Workowski et al., 2015). A major barrier to the prevention of morbidities is delayed diagnosis due to absence of or presence of mild non-specific symptoms (Wiesenfeld et al., 2005). Still, both clinical and subclinical upper tract inflammation can lead to chronic sequelae (Wiesenfeld et al., 2012). Laparoscopy with coincident biopsies of the endometrium and oviducts can confirm endometritis and salpingitis but this procedure is invasive and requires general anesthesia. Endometrial biopsy via suction catheter can be used to obtain tissue for use in the diagnosis of endometrial infection and inflammation associated with reduced fertility (Wiesenfeld et al., 2012). In fact, histologic endometritis is highly predictive of salpingitis and endometrial biopsy provides a specific diagnosis of PID (Kiviat et al., 1990). Although minimally invasive, the endometrial biopsy procedure is not routinely used for the diagnosis of endometritis because it requires trained clinicians and the biopsy procedure is invasive. A biomarker that identifies women with subclinical PID would significantly improve diagnosis and case management and could be used as a surrogate of upper reproductive tract disease for evaluation of STI therapeutics and vaccines (Poston et al., 2017).

We reported a distinct blood-derived mRNA profile in women with symptomatic PID and endometritis caused by GC and/or CT. Women with STI-induced endometritis exhibited enhanced expression of myeloid cell genes with suppression of genes involved in protein synthesis, mitochondrial oxidative phosphorylation, and T-cell responses compared to uninfected women or women with cervical infection only (Zheng et al., 2018). Women solely infected with CT expressed elevated levels of type I and type II interferon genes. The present study extends these findings by identifying a 21-gene blood biomarker that can aid prediction of STI-induced endometritis in symptomatic and asymptomatic women. Although insensitive for asymptomatic women with endometritis and low cervical burden, biomarker sensitivity for women with endometritis and high cervical burden was 83.3% (5/6). This biomarker distinguished women with STI-induced endometritis from patients with non-STI-induced pelvic pain, disseminated bacterial infections, lupus, and appendicitis, but failed to distinguish patients with tuberculosis, where a subset of the 21-gene set was also dysregulated.

## Methods

This study complied with the Declaration of Helsinki guidelines and all study participants provided written informed consent prior to initiation of study procedures. The Institutional Review Boards for Human Subject Research at the University of Pittsburgh, the University of North Carolina, and Johns Hopkins University approved the study.

### Study population

This study used whole blood collected from female participants recruited into three independent cohorts. The Anaerobes and Clearance of Endometritis (ACE) cohort was comprised of symptomatic women with clinically diagnosed PID, according to the Centers for Disease Control and Prevention diagnostic criteria (Workowski et al., 2015), who participated in a clinical trial (NCT01160640) comparing antibiotic regimens for PID treatment (Zheng et al., 2018). Diagnostic criteria included one or more of the following present on pelvic examination: cervical motion tenderness, or uterine tenderness or adnexal tenderness, in a sexually active young woman experiencing pelvic or lower abdominal pain (Workowski et al., 2015). The T cell Response Against Chlamydia (TRAC) cohort was comprised of asymptomatic women at high risk for STI (Russell et al., 2016). Both cohorts were recruited from clinics and emergency departments in Pittsburgh, PA. The Technology Enhanced Community Health-Nursing (TECH-N) cohort was comprised of women with clinical PID participating in a clinical trial of a nursing text-messaging intervention in Baltimore, MD (NCT01640379) (Trent et al., 2016).

### Data collection

As described previously for both ACE and TRAC, at enrollment, demographic data, a standardized medical history, and general physical and pelvic exams were performed. Cervical swabs were collected for microbiological molecular testing. Blood was collected for transcriptional profiling, and endometrial sampling was performed for microbiologic and histologic evaluation (Zheng et al., 2018); endometritis was defined according to published criteria (Kiviat et al., 1990). Chlamydial cervical burden was estimated via quantitative PCR using DNA extracted from reserved cervical swab eluates (Russell et al., 2016). The median cervical burden for the TRAC cohort = 10^4^ genome equivalents/swab (Russell et al., 2016); high and low burden were defined as values above and below this level, respectively. For patients in TECH-N, clinical, cervical microbiological, and blood transcriptional data were collected, but endometrial biopsy was not performed.

### Microarray data acquisition and processing

Total RNA was isolated from blood of TECH-N participants and analyzed via microarray (Illumina Human HT12 v3.0 expression bead chip) in the Genomics and Proteomics Core Laboratories at the University of Pittsburgh as described previously (Zheng et al., 2018) for ACE and TRAC participants and can be accessed from GEO (https://www.ncbi.nlm.nih.gov/geo/query/acc.cgi?acc=GSE110106). Transcripts were quantile normalized (Irizarry et al., 2003) and log2 transformed. Genes were filtered by expression (μ > 6.8) and variance thresholds (σ^2^ > 0.25). Batch effects were measured by guided PCA (Reese et al., 2013), and corrected using ComBat (Johnson et al., 2007). A total of 4952 transcripts passed initial filtering criteria. Array data associated with staphylococcal and streptococcal infections, lupus, appendicitis, and tuberculosis were retrieved from GEO (https://www.ncbi.nlm.nih.gov/geo/query/acc.cgi?acc=GSE19491; https://www.ncbi.nlm.nih.gov/geo/query/acc.cgi?acc=GSE83091) for evaluation of biomarker performance. The GSE19491 study was performed using Illumina HT V3 chips (~48,000 probes), our data (GSE110106) and the GSE83091 dataset were derived using Illumina HT V4 chips (~47,000 probes). The arrays share an overlapping set of ~40,000 probes that were used for cross-platform normalization. All data were quantile normalized and standardized.

### Study design

**Figure 1:**
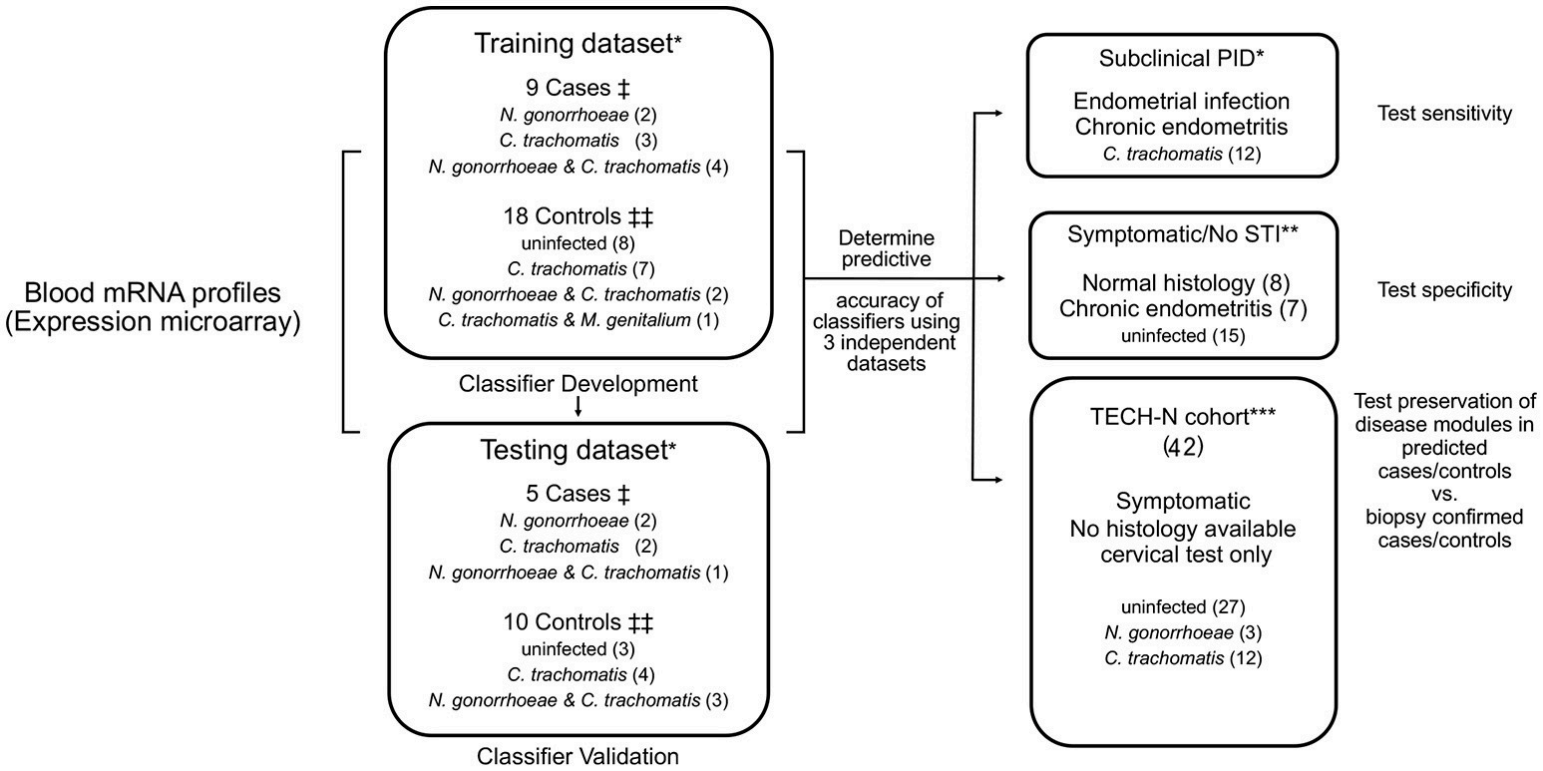
Diagram of study organization. Blood transcriptional mRNA profiles from cases and controls were analyzed in a training dataset. Identified classifier genes were subsequently validated in an independent testing dataset. The classifier genes were also evaluated in three additional independent patient datasets for sensitivity, specificity, and disease module preservation, respectively. ^†^Cases: women with symptoms consistent with PID and with biopsy confirmed endometrial STI with *N. gonorrhoeae* and/or *C. trachomatis* and/or *M. genitalium* and endometritis. ^‡‡^Controls: asymptomatic women who were uninfected or infected at the cervix only, with no endometrial inflammation. ^*^Participants recruited from TRAC cohort. ^**^Participants recruited from ACE cohort. ^***^Participants recruited from TECH-N cohort comprised women with symptoms of PID who had cervical microbiology determined but lacked endometrial data.

Cases were previously defined as women with a clinical PID diagnosis and biopsy-confirmed endometrial CT/GC (STI) and endometritis, and controls as study participants who did not display symptoms of PID, lacked endometrial infection and inflammation, and included women with cervical CT/GC and uninfected women (Zheng et al., 2018). For this study, two-thirds of cases and controls were assigned to a training dataset and the remaining one-third to a testing dataset based on the temporal order in which they were enrolled (Figure 1). The training dataset was used to derive a biomarker, that was then tested on the remaining cases and controls (testing dataset).

Study participants categorized as subclinical PID (*N* = 12) were asymptomatic with biopsy-confirmed endometrial CT/GC and endometritis. Symptomatic/No STI indicates women with clinical PID symptoms not infected with STI pathogens (*N* = 15). These independent groups were used to test the sensitivity and the specificity of the biomarker, respectively. An additional cohort of participants in TECH-N were used to test for preservation of disease modules (*N* = 42) (Figure 1).

Lastly, we tested the ability of the biomarker to discriminate STI-induced endometritis from other diseases including staphylococcal (*N* = 40) and streptococcal infections (*N* = 12), lupus (*N* = 28), appendicitis (*N* = 20), and tuberculosis (*N* = 82); and controls for staphylococcal and streptococcal infections (*N* = 23), controls for lupus (*N* = 17) and controls for tuberculosis (*N* = 40) using array data retrieved from GEO.

### Unsupervised hierarchical clustering, principal component analysis (PCA), classifier identification and validation

We applied unsupervised cluster analysis and PCA to identify reliable disease subgroups. For hierarchical clustering, genes were clustered via Pearson correlation, while samples were clustered using Spearman's rank correlation with average linkage criterion. To identify classifiers, a hybrid feature selection algorithm which combines two feature selection methods—the filter (moderate t statistics) and the wrapper (mSVM-RFE) was performed. The 4,952 transcripts that passed initial filtering criteria were used as a starting point for moderated *t*-tests using R package “limma”; genes with false discovery rate (FDR) < 0.05 and absolute fold change ≥1.35 were retained. A cut-off of 1.35 was based on reports that gene changes starting at 1.3-fold using microarray can be reliably confirmed by real-time PCR (Wurmbach et al., 2001; Zhou et al., 2007; Huggins et al., 2008). Next, Multiple Support Vector Machine Recursive Feature Elimination (mSVM-RFE) (Duan et al., 2005) was used to identify the smallest subset of features with maximal classification accuracy. SVM-RFE (Guyon et al., 2002) is an iterative algorithm that works backward from an initial set of features. At each round, it first fits a simple linear SVM, then ranks the features based on their weights in the SVM solution, and finally eliminates the feature with the lowest weight. mSVM-RFE extends this idea by using resampling techniques at each iteration to stabilize the feature rankings. We began by using the recursive procedure of Duan et al. (2005) to compute the ranking score statistics for each feature, then the top 100 ranked features were selected for further analysis. We used the ranks to define nested subsets of features F1⊂F2⊂…⊂F, and determined the best subset of features with smallest generalization error by varying a single parameter: the number of features. Multiple runs of fivefold cross-validation were used for estimation of generalization error.

mSVM-RFE identified the smallest subset of 21-gene with maximal classification accuracy. For prediction, SVM with 21 candidate biomarkers was used with the training dataset to generate a prediction model and to determine predictive accuracy in the testing dataset and three independent, disease-related, datasets. Data were standardized; linear kernel was used in SVM. With the predicted probability of the binary response (elevated risk of PID vs. low risk), a cut point of 0.5 was used. Validation of mRNA transcripts was performed by interrogating total RNA from participants via nCounter assay and were analyzed using NSolver v.3.0 software (Nanostring™). Differential expression between cases and controls was identified using *t*-test.

### Preservation module, gene set enrichment, and pathway analysis

Weighted Gene Coexpression Network Analysis (WGCNA) and a composite preservation statistic Z_summary_ (Langfelder et al., 2011) was used to determine if a module present in biopsy-confirmed cases was represented in biopsy-predicted TECH-N cases. Gene set enrichment analysis (GSEA) was performed to examine whether any preserved modules were significantly enriched in disease associated sectors generated by differential network analysis using WGCNA (Fuller et al., 2007; Luo et al., 2009; Zheng et al., 2018). Biological annotations of genes differing greatly between groups were explored with Ingenuity Pathway Analysis (IPA) (http://www.ingenuity.com/index.html). The significance of the canonical pathway was assessed using Fisher's exact test. Multiple testing was adjusted by Benjamini–Hochberg. All analyses were conducted in R (version 3.1.2).

## Results

### Training and testing dataset patient characteristics

Table 1 summarizes the distribution of demographic, clinical and microbiological characteristics of study participants assigned to training and testing datasets. Age, race, education, insurance, contraceptive status, bacterial vaginosis, or infection with *Trichomonas vaginalis* did not differ significantly between the cases and controls in either dataset (*p* > 0.05).

**Table 1:** Demographic, clinical, and microbiological characteristics of training and testing datasets.

	**Training dataset (*****N*** = **27)**			**Testing dataset (*****N*** = **15)**		
	**Case**	**Control**	**P value**	**Case**	**Control**	**P value**
**Number of subjects**	9	18		5	10	
**Age, y, median (range)**	23 (20–37)	21 (18–35)	0.288	21 (18–25)	19 (18–22)	0.117
**Race**			0.108			0.341
African American	3 (33)	10 (56)		5 (100)	6 (60)	
White	5 (56)	2 (11)		0 (0)	0 (0)	
American Indian/Alaska Native	0 (0)	1 (6)		0 (0)	0 (0)	
Multiracial	1 (11)	5 (28)		0 (0)	3 (30)	
Other	0 (0)	0 (0)		0 (0)	1 (10)	
**Ethnicity**			0.333			0.524
Hispanic or Latino	1 (11)	0 (0)		0 (0)	2 (20)	
Not Hispanic or Latino	8 (89)	18 (100)		5 (100)	8 (80)	
**Marital status**			0.628			1
Single	8 (89)	13 (72)		5 (100)	9 (90)	
Living with partner (at least 4 months)	1 (11)	5 (28)		0 (0)	1 (10)	
**Education level**			0.36			0.417
< HS graduate	2 (22)	3 (17)		0 (0)	2 (20)	
HS graduate of GED	2 (22)	8 (44)		3 (60)	4 (40)	
some college	2 (22)	6 (33)		1 (20)	4 (40)	
college graduate	2 (22)	1 (6)		0 (0)	0 (0)	
vocational	1 (12)	0 (0)		1 (20)	0 (0)	
**Insurance**			0.439			0.529
None	1 (11)	6 (33)		3 (60)	1 (10)	
Private	3 (33)	2 (11)		0 (0)	1 (10)	
Public	0 (0)	0 (0)		0 (0)	1 (10)	
Don't know	0 (0)	0 (0)		0 (0)	1 (10)	
Medicaid HMO	5 (56)	9 (50)		2 (40)	5 (50)	
Other	0 (0)	1 (6)		0 (0)	1 (10)	
***Trichomonas vaginalis***			0.268			1
Yes	0 (0)	4 (22)		0 (0)	0 (0)	
No	9 (100)	14 (78)		5 (100)	10 (100)	
**Bacterial vaginosis**			0.106			0.1305
Nugent Score 0–3	1 (11)	7 (39)		1 (20)	6 (60)	
Nugent Score 4–6	1 (11)	5 (28)		0 (0)	2 (20)	
Nugent Score 7–10	7 (78)	6 (33)		4 (80)	2 (20)	
**Contraception**						
Oral contraceptive pill			0.333			0.524
Yes	1 (11)	0 (0)		0 (0)	2 (20)	
No	8 (89)	18 (100)		5 (100)	8 (80)	
Depot medroxyprogesterone acetate			1			0.6
Yes	0 (0)	1 (6)		1 (20)	4 (40)	
No	9 (100)	17 (94)		4 (80)	6 (60)	
Intrauterine device			0.333			1
Yes	1 (11)	0 (0)		0 (0)	0 (0)	
No	8 (89)	18 (100)		5 (100)	10 (100)	
Condoms			0.695			1
Yes	4 (44)	10 (56)		4 (80)	7 (70)	
No	5 (56)	8 (44)		1 (20)	3 (30)	

### Transcriptional profiles define patients with STI-induced PID

**Figure 2:**
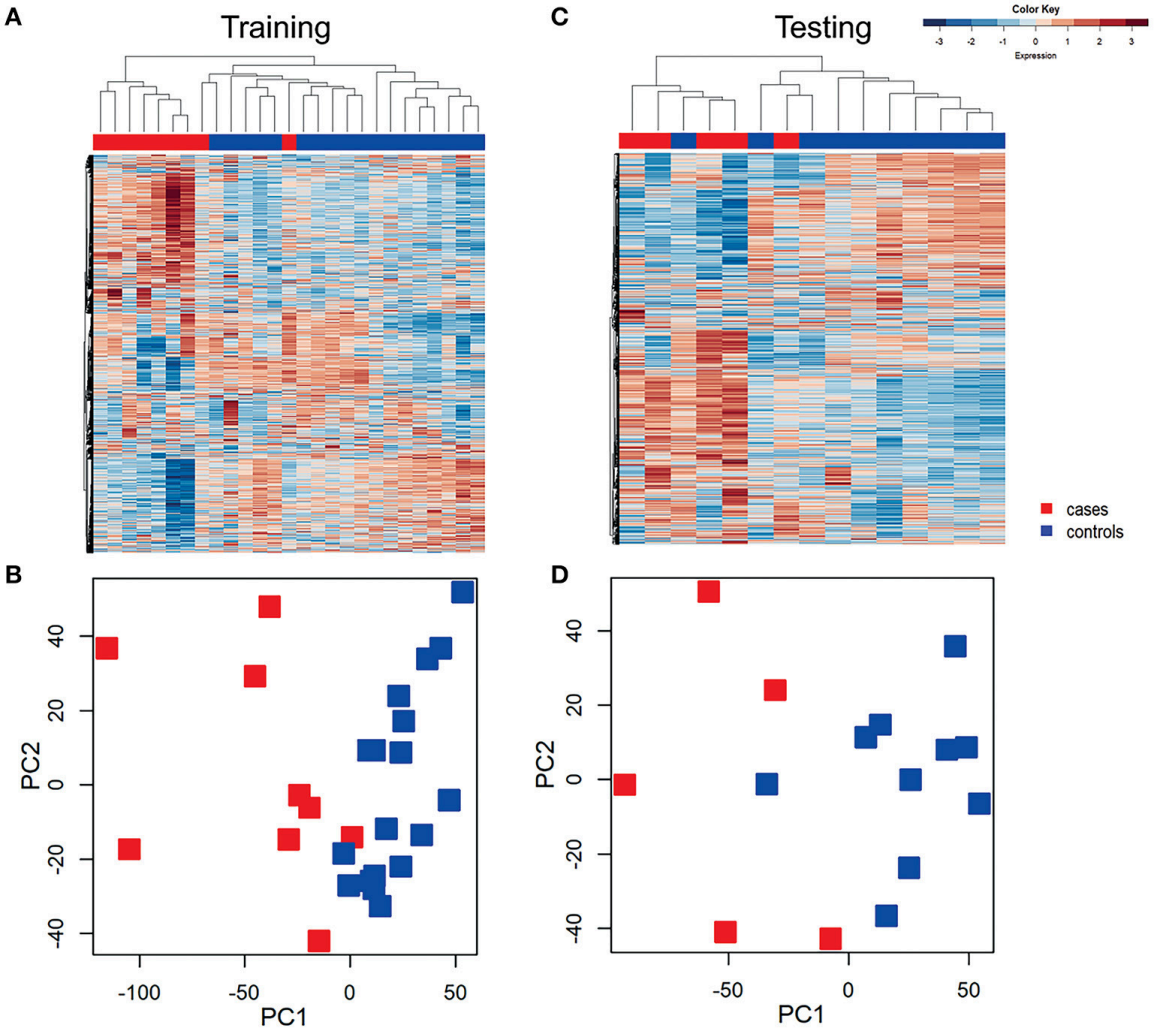
Unsupervised hierarchical clustering and principal component analysis (PCA) revealed distinctive blood mRNA transcriptional profiles separating cases from controls. Unsupervised hierarchical clustering of blood transcriptional profiles of women in training **(A)** and testing **(B)** datasets using 4952 transcripts. Samples were ordered by hierarchical clustering (Spearman correlation with average linkage) creating a condition tree, upper horizontal edge of heat map; study groupings (clinical phenotypes) are the colored blocks on the top of each profile. Heat map rows are genes; columns are participants. Principle component analysis of the variance in mRNA expression of the subjects depicted in the heat maps in training **(C)** and testing **(D)** datasets, using 4,952 transcripts and the same color scheme with each colored square depicting one subject. The x axis represents the first principal component, PC1, which accounts for the largest variance of mRNA expression, and the y axis, PC2, explains the second largest variance.

Unsupervised hierarchical clustering was conducted in training and testing datasets to determine if profiles grouped according to disease (Figure 2), and to visualize gene expression patterns. In the training dataset, transcriptional response profiles for 7 of 9 cases clustered tightly, and were easily distinguished from controls (*N* = 18). In the testing dataset, 4 out of 5 cases clustered and were distinct from 9 of 10 controls. Principal component analysis separated participants in a pattern consistent with the hierarchical clusters, with variance explained by the first principle component 61% and the second principle component 15%. These findings confirm that CT/GC-induced PID elicits a distinct blood transcriptional profile (Zheng et al., 2018).

### Classifier genes accurately diagnose women with STI-induced endometritis

**Figure 3:**
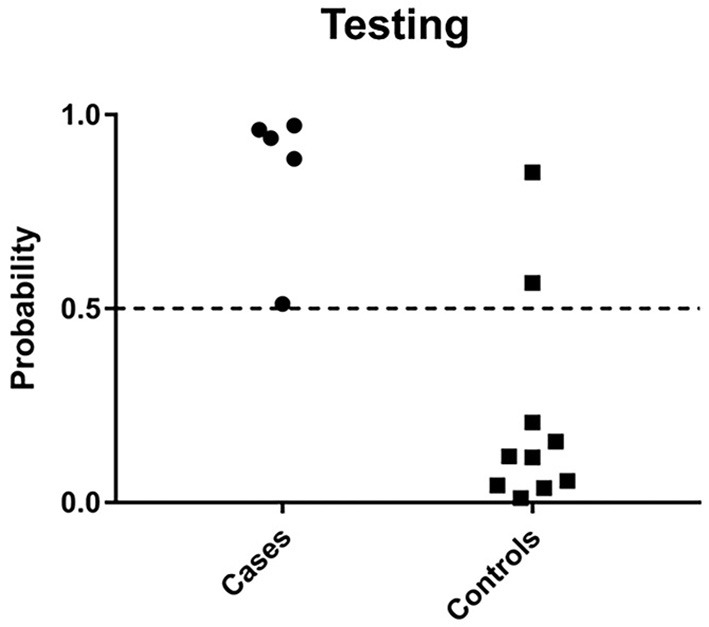
Predicted probability of STI-induced endometritis in testing datasets using a 21-gene signature and SVM learning algorithm. Each dot represents one subject. The x-axis indicates the biopsy confirmed groups of subjects and y-axis indicates the predicted probability of STI-induced endometritis. The dotted line corresponds to a predicted probability cutoff of 0.5. If the predicted probability of PID is > 0.5, we consider the predicted response as high risk.

Feature selection identified 21 genes (Table 2) that were used as classifiers in an SVM model and 10-fold cross-validation was conducted to determine their performance in the training dataset. Cross-validation achieved 100% accuracy, with 27 out of 27 patients correctly classified. Sensitivity, specificity, and overall predictive accuracy was 100% with the area under the receiver operating characteristic (ROC) curve = 1 (data not shown). We then determined the predictive performance of the panel in an independent testing dataset (Figure 3). Biomarker sensitivity was 100% (5 of 5 women with upper genital tract STI-induced endometritis were predicted as positive; 95% CI 80%, 100%), specificity was 80% (8 of 10 women who were negative for upper genital tract STI-induced endometritis by biopsy were predicted as negative; 95% CI 55%, 93%), and the overall predictive accuracy was 86.7% (13 of 15) with the area under the ROC curve = 0.96. Direction and magnitude of differential gene expression between cases and controls were validated for the training (*P* < 0.001 for all 21 genes) and testing datasets (*P* < 0.1 for all 21 genes) using nCounter expression array (Table 3).

**Table 2:** A list of 21 classifiers of STI PID using blood gene expression arrays.

**Gene**	**Adjusted *P*-value**	***P*-value**	**Fold change (case vs. control)**
**UPREGULATED**			
AIM2	1.31E-05	1.32E-08	2.06
DSC2	1.65E-05	3.02E-08	2.3
SIGLEC5	1.65E-05	2.81E-08	1.97
CASP1	1.87E-05	6.99E-08	1.58
CD58	1.87E-05	5.34E-08	1.53
GBP2	1.87E-05	7.90E-08	1.47
RNF19B	1.87E-05	8.15E-08	1.44
LIN7A	4.47E-05	5.87E-07	1.6
ADM	4.83E-05	7.22E-07	2.48
NAMPT	5.85E-05	1.08E-06	2.09
LOC440731	6.76E-05	1.48E-06	1.61
CYB5R4	6.88E-05	1.56E-06	1.46
PLAUR	9.56E-05	2.93E-06	1.56
FAS	0.00022	1.30E-05	1.39
LYSMD2	0.000698	8.78E-05	1.4
RPS15A	0.009756	0.0032	1.35
**DOWNREGULATED**			
FAIM3	1.87E-05	8.15E-08	−1.89
CD79A	3.33E-05	3.29E-07	−1.96
TCL1A	6.67E-05	1.41E-06	−1.85
EEF1D	0.000134	5.55E-06	−1.54
TSPAN3	0.001352	0.00025	−1.35

**Table 3:** Differential expression of 21 genes transcripts detected in blood using nCounter expression assay (NanoString Technologies) in training and testing datasets.

	**Pval**	**Fold change (case vs. control)**
**TRAINING DATASET**		
**Upregulated Genes**		
AIM2	5.54*E*−05	2.7
DSC2	6.78*E*−05	3.99
SIGLEC5	2.91*E*−06	2.75
CASP1	1.03*E*−04	1.81
CD58	5.55*E*−04	1.8
GBP2	1.14*E*−08	2.01
RNF19B	3.85*E*−07	1.74
LIN7A	1.40*E*−04	2.47
ADM	1.11*E*−05	3.74
NAMPT	5.87*E*−05	2.9
LOC440731	5.42*E*−04	2.98
CYB5R4	4.21*E*−04	1.8
PLAUR	2.59*E*−06	1.98
FAS	1.40*E*−06	1.72
LYSMD2	1.45*E*−04	1.57
RPS15A	4.29*E*−04	1.71
**Downregulated Genes**		
FAIM3	6.66*E*−04	−1.85
CD79A	7.66*E*−05	−2.2
TCL1A	3.50*E*−04	−2.62
EEF1D	2.26*E*−04	−1.26
TSPAN3	7.37*E*−05	−1.61
**TESTING DATASET**		
**Upregulated Genes**		
AIM2	0.0104	1.78
DSC2	0.004	3.25
SIGLEC5	0.0034	2.15
CASP1	0.0559	1.47
CD58	0.0001	1.84
GBP2	0.0649	1.48
RNF19B	0.0004	1.78
LIN7A	0.0001	2.99
ADM	0.0008	3.64
NAMPT	0.0001	3.05
LOC440731	0.0315	2.16
CYB5R4	0.0015	1.78
PLAUR	0.0008	2.03
FAS	0.028	1.49
LYSMD2	0.0634	1.45
RPS15A	0.0466	1.54
**Downregulated Genes**		
FAIM3	0.0564	−1.5
CD79A	0.0564	−1.53
TCL1A	0.0629	−1.65
EEF1D	0.0258	−1.25
TSPAN3	0.0243	−1.32

The 21-gene panel includes genes present in immune response pathways we reported as dysregulated in women with STI-induced endometritis (Zheng et al., 2018). The top up-regulated genes in the biomarker panel are involved in interferon signaling (AIM2, GBP2, CASP1, NAMPT, RNF19b), myeloid cell-mediated inflammation (ADM, LYSMD2, CD58, LIN7A, SIGLEC5, PLAUR), and cell death (DSC2, CYB5R4, FAS). The top down-regulated genes are involved in cell adhesion and migration (TSPAN3), protein synthesis (EEF1D), and lymphocyte signaling (TCL1A, CD79A, FAIM3).

### Utility of the biomarker for diagnosing subclinical STI-induced endometritis

**Figure 4:**
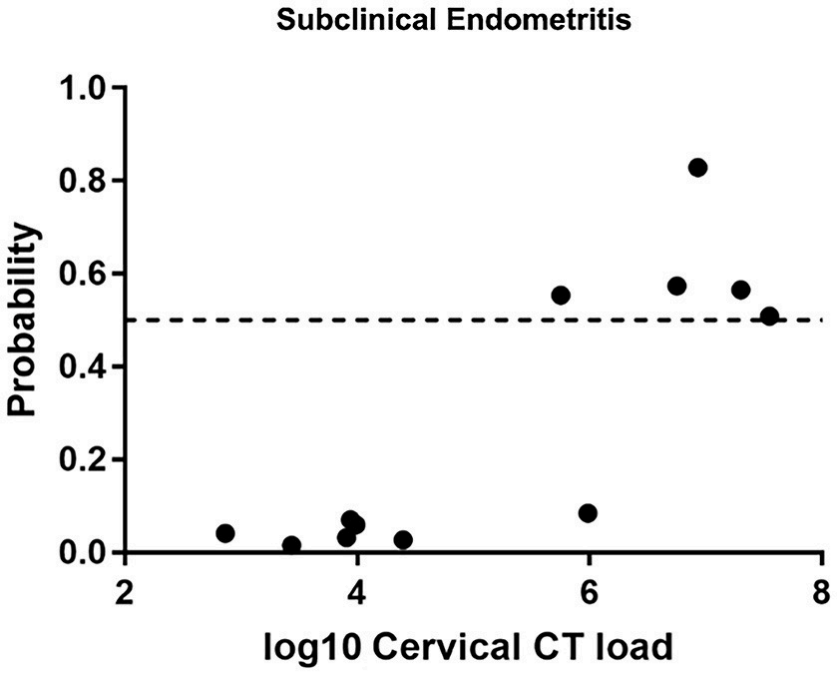
Predicted probability using 21-gene signature and SVM of STI-induced endometritis in a target dataset of asymptomatic subjects with biopsy proven subclinical CT/GC-induced endometritis. Each dot represents one subject. The x-axis indicates the log10 cervical *C. trachomatis* load and the y-axis indicates the predicted probability of STI-induced endometritis.

We evaluated the ability of the biomarker panel to diagnose subclinical STI-induced endometritis in asymptomatic women with endometrial infection and histologic evidence of endometritis (Figure 4). Among 12 asymptomatically infected women with endometritis, six had high cervical CT burden. The biomarker correctly identified STI-induced endometritis in 5 (83.3%). However, no women with low cervical CT burden were predicted positive. To determine if high cervical bacterial load drives the transcriptional signature, we compared gene expression levels in a group of women with normal histology and cervix-limited CT who differed only with respect to high (*N* = 5) vs. low burden (*N* = 11). No genes were significantly differentially expressed (FDR ≥ 0.3) after multiple comparison correction, revealing high CT burden was insufficient to drive the transcriptional signature. These data demonstrated the biomarker's ability to detect endometritis in women with elevated cervical CT burden, in the absence of symptoms.

### The biomarker can discriminate between STI-induced endometritis and pelvic pain without STI

**Figure 5:**
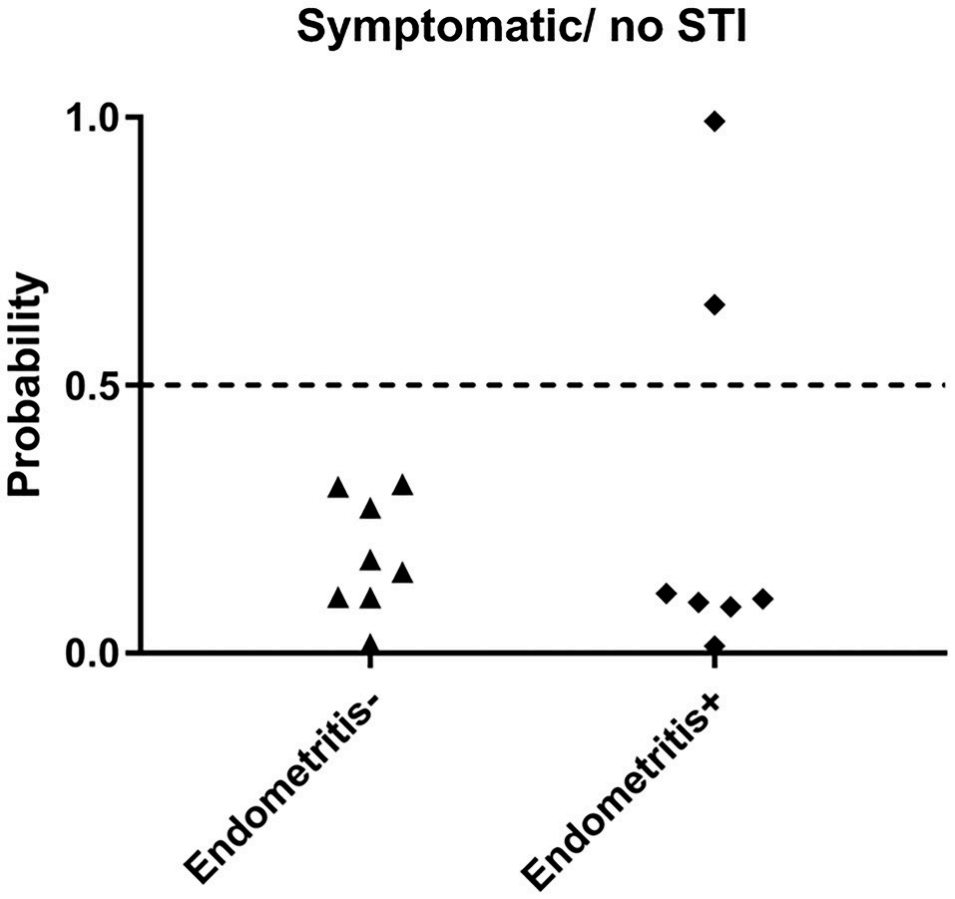
Predicted probability using 21-gene signature and SVM of STI-induced endometritis in a dataset of women who were symptomatic but were uninfected. Subsets of patients within the dataset were positive and negative for histologic endometritis. Each dot represents one subject. The x-axis indicates two groups of women with pelvic pain without STI. One group has normal histology, the other group has chronic endometritis; y-axis indicates the predicted probability of STI-induced endometritis.

Next, we evaluated the biomarker in a group of women with pelvic pain and tenderness consistent with a PID diagnosis (Workowski et al., 2015) but without STI. Endometrial biopsies revealed normal histology (*n* = 8) or chronic endometritis (*n* = 7) (Figure 5). All 8 women with pelvic pain and normal histology were predicted negative (100% specific), and 5 of 7 uninfected women with pelvic pain and chronic endometritis due to undetermined cause were predicted negative (71.4% specific). These data demonstrated the ability of the biomarker to discriminate women with STI-induced endometritis with 86.7% specificity overall.

### STI-induced endometritis modules are preserved in a cohort of women predicted positive by the biomarker

The biomarker was evaluated in 42 TECH-N study participants (Trent et al., 2016). All TECH-N participants were diagnosed with clinical PID; 15 had documented CT and/or GC infection. Almost all participants were African American (98%) with a median age of 19 years. Of the 42 women, 23.8% were predicted positive for STI-induced endometritis. Among the 15 women with cervical STI, 6 (40%) were predicted positive: 3 of 3 with GC infection (100%) and 3 of 12 with CT (25%). In contrast, 4 of 27 women without STI (14.8%) were predicted positive.

**Figure 6:**
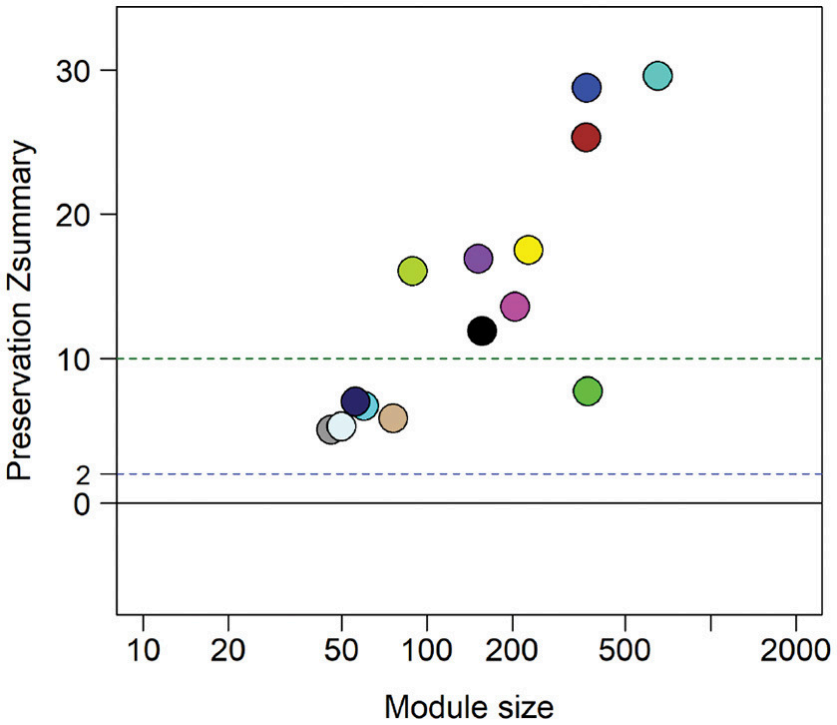
Zsummary statistics reveal module preservation of blood profiles from women with biopsy-confirmed CT/GC-induced endometritis compared to women with biomarker-predicted STI-induced endometritis from independent TECH-N cohort. Colored circles correspond with highly correlated gene modules identified in biopsy-confirmed CT/GC-induced endometritis. The x-axis indicates the number of genes in each module; y axis is the preservation Zsummary value. The dotted lines with cutoff Zsummary of 2 and 10 indicate preservation and high level preservation respectively.

The absence of upper genital tract microbiological and histological data for this cohort prevented direct evaluation of biomarker accuracy. However, we performed an indirect assessment by determining if the gene expression profiles of the 8 predicted cases showed conservation of 14 module networks (highly correlated genes) identified in 14 biopsy-confirmed cases (Zheng et al., 2018). Preservation analysis revealed that 8 modules yielded a zSummary statistic > 10, reflecting strong conservation (Figure 6). We also performed differential network analysis using WGCNA to detect significant and concordant differences between biopsy-confirmed cases and controls and between predicted cases and controls (Table 4, significance of module enrichment in sectors determined by GSEA). Five modules were differentially expressed (FDR < 0.05, Table 4) between combined sets of biopsy-confirmed cases (*n* = 14) and controls (*n* = 28). The top 4 modules were also differentially expressed (FDR < 0.05, Table 4) between predicted TECH-N cases (*n* = 8) and predicted controls (*n* = 23). Functional annotation of these modules (Table 4) was performed as before using IPA. STI-induced endometritis-associated genes and activated pathways detailed previously were replicated with overexpression of genes in myeloid cell and NF-κB activation pathways and integrin-linked kinase signaling; and suppression of T cell specific genes, and genes for protein synthesis and mitochondrial oxidative phosphorylation (Zheng et al., 2018). These findings indicated that the predicted cases recapitulated the STI-induced endometritis profile of biopsy-confirmed cases, providing further evidence that the biomarker reflects gene changes that occur in patients with STI-induced endometritis.

**Table 4:** Preservation and functional annotation of significantly differentially expressed modules between cases and controls by Gene Set Enrichment Analysis (GSEA).

**Module**	**Preservation Zsummary**	FDR^*^(biopsy-confirmed cases vs. controls)	**FDR (predicted cases from Tech-N vs. controls)**	**Enriched ingenuity canonical pathways**
Turquoise	32	1.58E-84	5.75E-15	Overexpression of myeloid cell genes
Yellow	20	2.47E-34	6.29E-14	Suppression of T-cell specific genes
Brown	28	4.07E-21	3.55E-10	Suppression of protein synthesis and mitochondrial oxidative phosphorylation
Midnight Blue	8	3.64E-06	2.46E-02	Overexpression of NF-κB and Integrin-linked kinase signaling
Purple	19	1.76E-03	^***^NS	Suppression of cell cycle control and regulation

* *FDR, False discovery rate*;

*** *NS, Not significant (FDR > 0.05)*.

### The biomarker distinguishes STI-induced endometritis from other infectious and inflammatory conditions

**Figure 7:**
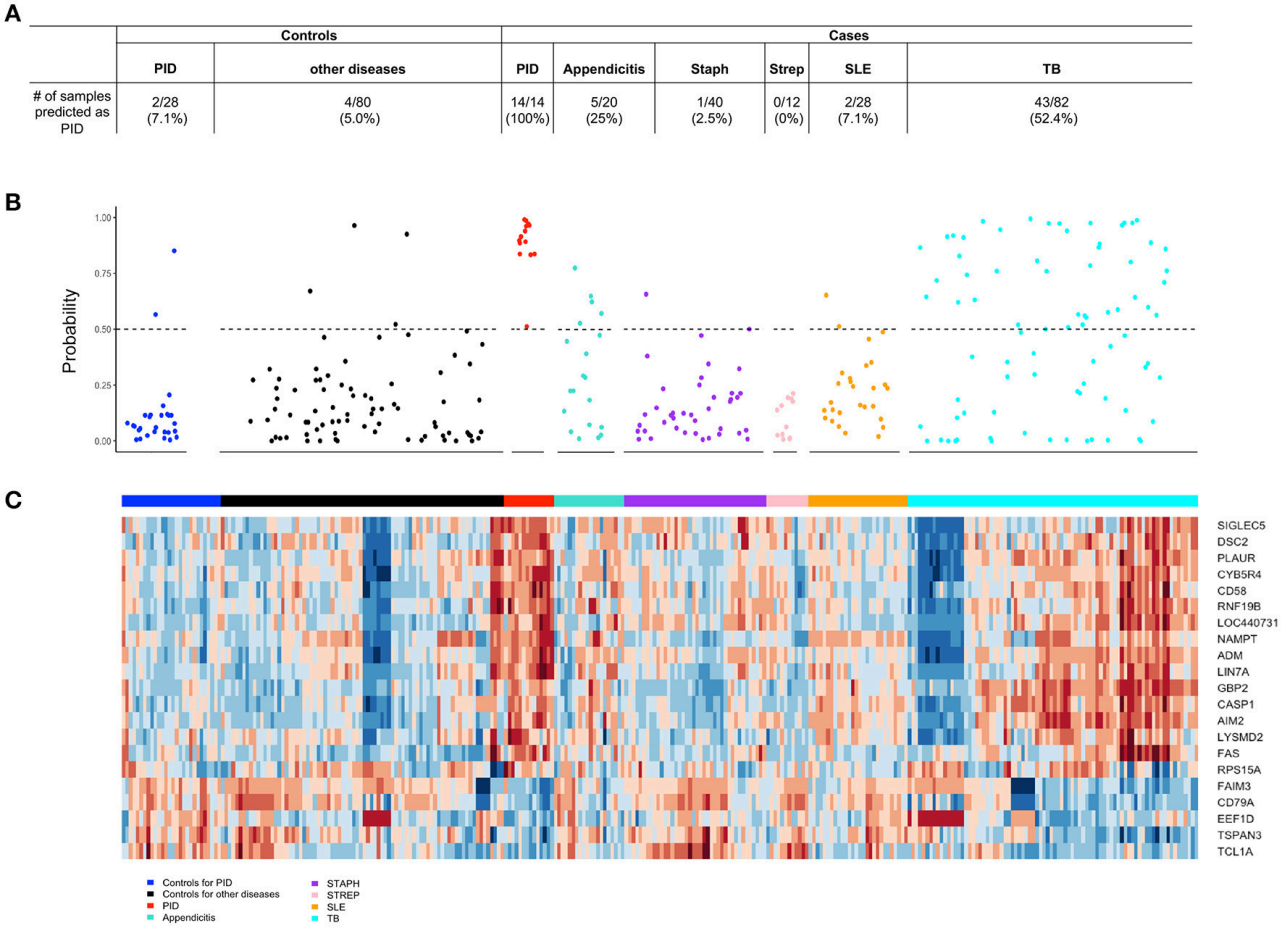
The 21-gene biomarker distinguishes STI-induced endometritis from other infectious and inflammatory conditions. **(A)** The number of samples within each disease and control group predicted as STI-induced endometritis is summarized in the table. **(B)** The predicted probability of STI-induced endometritis in each disease and control group using a 21-gene biomarker. Each dot represents one subject. The x-axis indicates the groups of subjects and y-axis indicates the predicted probability of STI-induced endometritis. The dotted line corresponds to a predicted probability cutoff of 0.5. **(C)** Unsupervised hierarchical clustering using 21-genes revealed distinctive blood mRNA transcriptional profiles separating STI-induced endometritis from other diseases and controls. Study groupings (clinical phenotypes) are the colored blocks on the top of each profile. Heat map rows are genes; columns are participants. Transformed expression levels are indicated by color scale, with red representing relative high expression and blue relative low expression.

The differential diagnosis of PID includes pain due to endometriosis, appendicitis, urinary tract infection, ovarian torsion, interstitial cystitis, and, less commonly, adnexal tumors. Of these diseases, transcriptional profiles were only available for appendicitis. The biomarker correctly identified 15 of 20 appendicitis patients (75%) as negative for STI-induced endometritis. Interestingly, patients with tuberculosis displayed substantial change compared to their healthy controls leading to 52.44% (43 out of 82) being wrongly identified as STI-induced endometritis. Accuracy was improved for patients with systemic infections caused by *Staphylococcus* (97.5%; 1 out of 40) or *Streptococcus* (100%; 0 out of 12), and for patients with lupus (96.4%; 2 out of 28); these patients showed minimal or no change in the biomarker compared to their healthy controls (Figure 7).

To determine overlapping response pathways between patients with STI-induced endometritis and those with appendicitis, we accessed whole blood gene expression profiles published in GEO of male and female patients with appendicitis and others with non-specific abdominal pain who served as controls (Chawla et al., 2016). Using IPA, we performed functional annotation of genes that were differentially expressed between these groups as determined by a moderated *t*-test. We compared the top 10 up- or down-regulated pathways in appendicitis patients and women with PID vs. each group's controls (Tables 5, 6). The top up-regulated pathway in women with STI-induced endometritis was interferon signaling, which was down-regulated in patients with appendicitis. Pathways related to myeloid cell activation were up-regulated in both disease states. Pathways enriched in mitochondrial genes and genes for protein synthesis were down-regulated for both patient groups. T cell signaling pathway genes were significantly down-regulated in STI-induced endometritis patients only.

**Table 5:** Top 10 up-regulated pathways in acute appendicitis patients vs. controls with non-specific abdominal pain.

**Canonical pathways**	*P*-value^*^	FDR (BH)^**^	Ratio^***^	**Molecules**
3-phosphoinositide biosynthesis	1.51E-05	0.001778	0.1	PPFIBP2, PIP4K2B, PPP4R1, HACD2, PTPN12, UBLCP1, SACM1L, PTEN, MTMR6, PPP1CC, MTMR4, SYNJ1, PPP1R12A, DUSP1, PIK3CG, PPM1F, PIK3AP1, RNGTT, PTPN22, ATM
Production of nitric oxide and reactive oxygen species in macrophages	3.31E-05	0.001778	0.0979	RAP1B, MAP3K1, IFNGR1, NCF4, JAK2, IRF1, TLR2, PPP1CC, LYZ, RHOQ, RHOB, PPP1R12A, PIK3CG, CAT, NCF2, CYBB, MAP3K2, ATM, PRKCA
Inflammasome pathway	3.72E-05	0.001778	0.3	IL18, NLRP3, AIM2, CASP1, CTSB, CASP5
ILK signaling	4.07E-05	0.001778	0.0964	PXN, PDPK1, VIM, HIF1A, CREB5, PTEN, NCK2, MYC, RHOQ, RHOB, PPP1R12A, LIMS1, CDC42, PIK3CG, CREB1, RSU1, PTGS2, ITGB5, ATM
Role of pattern recognition receptors in recognition of bacteria and viruses	6.17E-05	0.002455	0.109	NLRP3, RNASEL, TLR2, IFIH1, CLEC7A, IL18, TLR5, PIK3CG, CREB1, TLR1, CASP1, OSM, EIF2AK2, ATM, PRKCA
PI3K/AKT signaling	8.32E-05	0.00309	0.112	YWHAG, ITGA5, PDPK1, JAK2, PTEN, YWHAQ, HSP90B1, SYNJ1, LIMS1, PIK3CG, HSP90AA1, CDKN1B, PTGS2, MCL1
TREM1 signaling	0.000204	0.005754	0.133	TLR2, TREM1, IL18, NLRP3, TLR5, TLR1, CASP1, ITGA5, JAK2, CASP5
Fcγ receptor-mediated phagocytosis in macrophages and monocytes	0.000295	0.007413	0.118	NCK2, PXN, ACTR3, CBL, FCGR2A, CDC42, PIK3CG, ARPC5, FYB1, PRKCA, PTEN
Integrin signaling	0.000479	0.010715	0.0822	RAP1B, PXN, ASAP1, ARPC5, ITGA5, PTEN, NCK2, WIPF1, RHOQ, ACTR3, RHOB, PPP1R12A, LIMS1, CDC42, PIK3CG, NEDD9, ITGB5, ATM
Phagosome maturation	0.010715	0.010715	0.0946	TUBB1, DCTN4, VPS41, CTSO, TUBB2A, TUBA4A, ATP6V1A, LAMP2, CTSH, HLA-DRB3, NCF2, CYBB, CTSB, CTSC

* *P-value: The p value identifies statistically significant over-representation of focus genes in a given process. Over represented functional or pathway processes are processes which have more focus genes than expected by chance*.

** *False Discovery Rate by Benjamini–Hochberg procedure*.

*** *Ratio: The ratio indicates the number of differentially expressed genes that map to the pathway divided by the total number of genes that map to the canonical pathway*.

**Table 6:** Top 10 down-regulated pathways in acute appendicitis patients vs. controls with non-specific abdominal pain.

**Canonical pathways**	*P*-value^*^	FDR (BH)^**^	Ratio^***^	**Molecules**
Mitochondrial dysfunction	0.000145	0.016982	0.0468	FIS1, COX17, UCP2, ATP5D, UQCRH, NDUFB7, NDUFA12, NDUFA3
Systemic lupus erythematosus signaling	0.000182	0.016982	0.04	CD3E, IL1RN, SNRPF, IL6R, SNRPB2, SNRNP70, PLCG1, CD79A, HLA-F
Calcium-induced T lymphocyte apoptosis	0.000295	0.019498	0.0758	CD3E, ZAP70, PLCG1, ATP2A3, EP300
Remodeling of epithelial adherens junctions	0.000363	0.019498	0.0725	RAB5C, ARPC1B, TUBA1C, TUBB, ACTG1
Oxidative phosphorylation	0.000417	0.019498	0.055	COX17, ATP5D, UQCRH, NDUFB7, NDUFA12, NDUFA3
CTLA4 signaling in cytotoxic T lymphocytes	0.001862	0.066069	0.0505	FYN, AP2M1, CD3E, ZAP70, PLCG1
Interferon signaling	0.003548	0.100462	0.0833	IFI6, PSMB8, BAX
EIF2 signaling	0.003548	0.100462	0.0317	RPL4, RPS26, RPL17, RPS15A, RPS29, RPL10A, RPLP0
Caveolar-mediated endocytosis signaling	0.003631	0.100462	0.0563	FYN, RAB5C, ACTG1, ITGAX
Antigen presentation pathway	0.004571	0.10617	0.0789	PSMB8, CD74, HLA-F

** *False Discovery Rate by Benjamini-Hochberg procedure*.

* *P-value: The p value identifies statistically significant over-representation of focus genes in a given process. Over represented functional or pathway processes are processes which have more focus genes than expected by chance*.

*** *Ratio: The ratio indicates the number of differentially expressed genes that map to the pathway divided by the total number of genes that map to the canonical pathway*.

## Discussion

We previously characterized distinct blood mRNA profiles in STI-induced endometritis (Zheng et al., 2018) that indicated a pathway for biomarker discovery. We have extended our findings by generating a biomarker that can identify women at high risk for STI-induced endometritis. We identified a 21 gene-panel that accurately identified symptomatic women with upper genital tract CT/GC and inflammation. This panel also accurately identified asymptomatic women with cervix-limited infection and uninfected women as controls, suggesting that the biomarker is not driven by cervical infection. We were able to determine STI-induced endometritis with 83.3% accuracy in asymptomatic women with high cervical CT burden. This demonstrates the ability of the biomarker to detect women with CT-induced, subclinical endometritis but the panel failed to identify women with low cervical burden who had endometritis, indicating that diagnostic sensitivity is positively associated with bacterial load. This suggests that the biomarker identifies women experiencing hyperinflammation (peak illness), while mild or moderate inflammation (initial or recovery phase) may be undetectable. This lack of sensitivity likely reflects restriction of CT infection to the reproductive tract mucosa. Since over 90% of CT infections are asymptomatic in women, identification of enhanced risk for endometritis in any infected woman, regardless of burden, would enhance clinical care by providing the patient with information related to their risk of long-term sequelae.

The biomarker correctly identified 100% of women with pelvic pain without infection and without endometritis as negative. Two of 7 STI-uninfected symptomatic women with chronic endometritis were predicted as cases. These misclassifications could reflect PID from infection by microorganisms not evaluated here (Haggerty et al., 2016) or recent resolution of STI before enrollment with residual endometritis. Six of seven had documented GC or CT infection history and two reported prior PID. In addition, using an independent cohort of women with clinically diagnosed PID and cervical CT/GC, the biomarker predicted 40% to have endometritis. This percentage is consistent with morbidity rates after PID (Price et al., 2013). Furthermore, disease-related modules differentially expressed in biopsy-confirmed cases and controls were also differentially expressed in biomarker-predicted cases and controls.

The biomarker successfully distinguished women with STI-induced endometritis from patients with streptococcal or staphylococcal infection, and lupus. Examination of pre-defined blood modular signatures has revealed that patients with lupus and streptococcal infection do not exhibit elevated inflammation transcripts (Berry et al., 2010) while patients with staphylococcal infection demonstrated enhanced inflammation transcripts but minimal change in IFN-inducible transcripts, and B- and T cell transcripts. STI-induced endometritis contrasted with all groups, characterized by enhanced inflammatory transcripts, including IFN-responsive genes, with significantly decreased T cell transcripts (Zheng et al., 2018). However, the biomarker was unable to reliably distinguish tuberculosis from STI-induced endometritis. The blood-derived modular signature of tuberculosis was paralleled in STI-induced endometritis, including enhanced IFN-inducible genes, myeloid inflammatory response genes, and decreased T cell transcripts. Fortunately, our biomarker was quite accurate at distinguishing STI-induced endometritis from appendicitis, which is in the differential diagnosis. The decrease in interferon signaling genes in appendicitis patients likely provides increased specificity. In sexually active females presenting with abdominal pain, a negative result with the biomarker would suggest possible appendicitis rather than endometritis.

Limitations of our study included small sample size and absence of longitudinal data regarding development of reproductive tract sequelae in women predicted to have STI-induced endometritis. A large independent cohort is warranted to validate the sensitivity and specificity. The biomarker was unable to detect women with endometritis who had low cervical bacterial burden, indicating the potential to miss women at risk for sequelae who may be in the early phases of infection and inflammation, or are in a resolution phase.

PID diagnosis is based on clinical criteria and confounded by wide variation in symptoms. Furthermore, asymptomatic CT/GC-infected women may sustain endometrial infection and damaging inflammation (Wiesenfeld et al., 2012). No single test or combination of diagnostic indicators reliably identifies endometritis. The most specific criteria currently available include endometrial biopsy with histopathologic evidence of endometritis, transvaginal sonography or magnetic resonance imaging showing thickened, fluid-filled tubes with or without free pelvic fluid or tubo-ovarian complex, or Doppler studies suggesting pelvic infection (e.g., tubal hyperemia); or laparoscopic findings consistent with PID, although this also requires endometrial biopsy if no visual evidence of salpingitis is present (Workowski et al., 2015). Although endometrial biopsies can be performed with a suction pipelle, this procedure is difficult to perform routinely in the clinic, and laparoscopies require general anesthesia.

The nCounter Dx Analysis system (NanoString) used to confirm the mRNA results in this study, provides rapid, reliable, inexpensive, and reproducible molecular sub-grouping of clinical samples (Northcott et al., 2012; Veldman-Jones et al., 2015; Masucci et al., 2016). Results of the assay can easily be available within 24–48 h of obtaining blood from the patient. A blood biomarker that informs the clinician and the patient that the patient has upper genital tract inflammation coincident with their lower genital tract STI indicates increased risk for long term sequelae. This knowledge may prompt more intensive screening for STIs in that patient, with a goal to prevent long-term sequelae. A biomarker of STI-induced endometritis may also be used to monitor the benefit of novel therapies ancillary to antibiotic treatment, such as steroids or other anti-inflammatory medications, or as an end point to evaluate vaccines. Newer larger cohort and longitudinal studies that include evaluation of long-term sequelae are warranted to validate this biomarker.

## Author contributions

XJ designed the study and prepared the manuscript. CO designed the study and prepared the manuscript; She also oversaw preparation of specimens for analysis. WZ assisted with biostatistical analysis. TP and BT contributed to the preparation of the final manuscript submission. HW, SH, MT, and CG contributed to recruitment, clinical evaluation and diagnosis of study participants. GT assisted with biostatistical analysis. TD designed the study and prepared the manuscript.

### Conflict of interest statement

The authors declare that the research was conducted in the absence of any commercial or financial relationships that could be construed as a potential conflict of interest.
